# Memories of being injured and patients' care trajectory after physical trauma

**DOI:** 10.1186/1472-6955-7-8

**Published:** 2008-06-17

**Authors:** Mona Ringdal, Kaety Plos, Ingegerd Bergbom

**Affiliations:** 1 Institute of Health and Care Sciences, The Sahlgrenska Academy, University of Gothenburg, Box 457, S-40530 Gothenburg, Sweden

## Abstract

**Background:**

The purpose of this study was to acquire a deeper understanding of patients' memories of being injured and the trajectory of care before, during and after their Intensive Care Unit (ICU) stay.

**Methods:**

Interviews were conducted with eighteen informants who after physical trauma had been cared for in the ICU. The interviews were analyzed by using a phenomenological hermeneutical method.

**Results:**

The memories of injury during the trajectory of care are illustrated in a figure in which the injured informants have memories from five scenes; the scene of the accident, emergency unit, ICU, nursing ward and of coming home. Twelve subthemes were abstracted and four themes emerged; a surrealistic world, an injured body, care, and gratitude for life. After the accident, a "surrealistic world" appeared along with bad memories of being in a floating existence where plans had to be changed. This world was unfamiliar, sometimes including delusional and fragmentary memories from the ICU, and it was experienced as uncontrollable. They felt connected to an "injured body", experiencing bad memories from the ICU of being injured, from the nursing ward of simply enduring and of being in a No Man's Land when coming home; their lives had become limited. At the same time they were "connected to care" with good memories of receiving attention from others at the scene of the accident, being taken cared of at the emergency unit and cared for in the ICU. This care made them realise that people are responsible for each other, and they felt comforted but also vulnerable. Finally, they experienced "gratitude for life". This included good memories of being loved together with support from their families at the ICU, wanting to win life back at the nursing ward and acceptance when returning home. The support from their families made them realise that they fit in just as they are.

**Conclusion:**

When bad memories of a surrealistic world and of being injured are balanced by good ones of care and love with a gratitude for life, there are more possibilities to move on despite an uncertain future following the injury.

## Background

Memories of being cared for in connection with injury are probably extremely important for most people [1]. Memories of important events often affect people in one way or another. The episodic memory makes it possible for persons to mentally travel back in time and remember what was experienced [2]. When remembering a traumatic event, people often separate memories of life into before and after. It is more than just a trauma and becomes a starting point of a journey [3]. After an injury, people can have memories of losing control of the situation, feeling pain and being dependent on assistance of others which can be extremely stressful [4]. In the Intensive Care Unit (ICU), the treatment and environment contribute to delusional, fragmentary memories and amnesia [5]. Patients with traumas in the ICU who report delusional memories, also report more unexplained feelings of panic after discharge [6]. After the ICU stay, patients need help piecing together events from their time in hospital [7]. Relatives may provide an important link to reality and the patients' life [8]. This can help the survivors of a trauma to create a history of what has happened. The impact of the critical condition can be apparent in the patients' lives post-discharge even if there is a lack of memories [9]. The process of finding meaning in what has happened, and of understanding and living with fragmentary memories, is a challenge to patients that suffer from an injury. The theory of responding to threats to integrity of self [10,11] describes the importance of preserving the self and the work of regaining the self. What is not clear is how fragmentary, delusional memories or amnesia in connection with injury and care influence the patients' lives. The purpose of this study was to acquire a deeper understanding of patients' memories of being injured and the trajectory of care before, during and after their ICU stay.

## Methods

The inductive method of phenomenological hermeneutics, mainly inspired by Ricoeur [12] and described as a method by Lindseth & Norberg [13], was used to reveal perceptions and interpretations from the informants' narratives about their memories of an injury and the care afterwards. The interview text was condensed and abstracted into subthemes and themes and finally interpreted as a whole in a comprehensive understanding which discloses new possibilities for being in the world. The method focuses on interpretation, and the pre-understanding will contribute to a first orientation of the text. The understanding of the text will then challenge the current pre-understanding which in turn will lead to new questions and further understanding of the phenomena [14].

### Selection of informants

We used a purposeful and convenient sampling approach [15], seeking information from informants with different memories (factual, delusional, fragmentary memories or amnesia) following a trauma in order to understand the range of memories and the extent of the phenomenon. A selection of informants was obtained from the data from a previous study with 239 patients > 18 years who were treated for trauma in the ICU and who related their experiences of memories from there. Exclusion criteria were; suicide attempt, intellectual impairment, not resident in Sweden, unknown address [6]. In this previous study, 61 patients stated in the questionnaire that they experienced delusional memories, and 27 patients experienced amnesia or fragmentary and factual memories but no delusional memories during the ICU stay. For the present study we invited ten informants from each group. The informants came from different ICUs in an area of Sweden with about 1500 000 inhabitants. The intention was to reach informants of different age, gender, traumas, memories and time in the ICU. The informants were not intended to represent all patients with trauma, but rather to help us gain a perspective on the range of injured people's perspective on their memories of being injured and cared for. We sent them a letter including written information about the study and the need for informed consent, inviting them to participate. Telephone contact with the informants was established one week after the letter was sent to obtain verbal informed consent and to arrange a suitable time for an interview. Eighteen out of twenty invited informants chose to participate and two declined participation. The studied sample consisted of nine men and nine women from four different hospitals; one university hospital (n = 11), two county hospitals (n = 6) and one district hospital (n = 1). Fourteen of the informants had been injured in traffic accidents, one had fallen from a great height, two had suffered occupational injury and one recreational injury. The median age was 48 years (range 22 to 67 years); the median age for women was 40 years and 54 years for the men. Fifteen informants (eight men and seven women) worked before the injury occurred, two were retired and one was on sick leave. Nine (five men and four women) had returned to full or part time work at the time of the interview. Seven of them lived alone. Thirteen informants suffered from multiple traumas, two had an isolated head injury trauma and three had unspecific smaller traumas. The median injury severity score (ISS) [16] was 5 (range 1–29) for the women and 17 (range 4–29) for the men. All of the informants were cared for in the ICU for a median period of 5.5 days (range 1–38 days). Five women and seven men were on mechanical ventilation for a median period of 2 days (range 1–37 days).

### Interviews

One of our team (MR) conducted the interviews from April 2004 to September 2004, 20–36 months after the occurrence of the injury. The informants chose the location of the interview and it took place either in the informants' home (n = 11), in a quiet room at the university (n = 6) or at the hospital (n = 1). The interviews lasted approximately 60–120 minutes and were audio taped. The interview was initiated with an open question, where the informants were requested to narrate their memories of being injured in an accident and cared for in the hospital. In order to elucidate or receive explanations from the informants' stories, additional questions were asked, for example: What were your feelings? Please explain. The informants received verbal and written information about the purpose and procedure of the study and gave verbal consent to participate. The informants were assured confidentiality and were informed that they could withdraw from the study at any time. The Research Ethics Committee of the University of Gothenburg approved the study.

### Data analysis

We transcribed the interviews verbatim and analyzed them with a phenomenological hermeneutical method. The interview text consisted of 171 pages of extensive and rich information. The analysis, based on principles described by Lindseth & Norberg [13], began with a naïve reading by which we transformed the text from being natural to a pervading phenomenological approach. This was validated by several structure analyses. In the first structure analysis the whole text was read again and divided into meaning units. It was then condensed and abstracted into subthemes and set in chronological order. This describes the informants' memories during the trajectory of care before, during and after the ICU.

The second structure analysis describes in what ways the different subthemes were connected to each other and assembled into themes. The comprehensive understanding finally describes new possibilities for living with memories of an injury during the trajectory of care. The naïve reading for each interview as well as the first and second structure analysis was carried out by two members of the theme (MR, IB). All members of the theme (MR, KP, IB) read and wrote the text. The first author has experience as a critical care nurse, the second and the third as researchers within the field of intensive care.

## Results

All informants told their stories in a vivid and in-depth way. Ten informants had both factual and delusional memories from the ICU. Five informants had fragmentary memories of the first days after the injury. Three informants had amnesia for several weeks after the injury. They narrated their experiences of being without memory of the injury and what that had meant to them after coming home.

Based on the naïve reading, patients' memories before, during and after the ICU stay were abstracted from the meaning units into subthemes during the trajectory of care with five chronological scenes; the scene of the accident, the emergency unit, the ICU, the nursing ward and the coming home. In the first structure analysis we identified two to four subthemes from each scene of care (Table 1). Note that verbatim comments are used to illustrate issues and themes. Participants were assigned numbers to maintain confidentiality.

**Table 1:** First structure analysis, subthemes during trajectory of care before during and after ICU stay.

**Scene of the accident**	**Emergency unit**	**Intensive care unit**	**Nursing ward**	**Coming home**
To be in a floating existence	Changes of plans	To be injured	To be enduring	To be in No Man's Land
To receive attention	To be taken care of	To be in an unreal world	To win life back	To be in acceptance
		To be cared for		
		To be loved		

**Figure 1:**
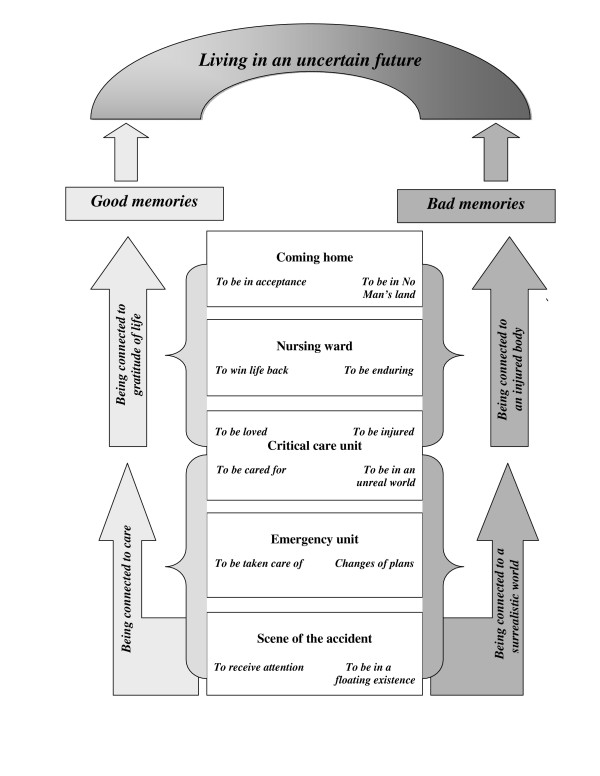
**The trajectory of care after injury.** Good memories balancing bad ones in an uncertain future.

In the second structure analysis all the twelve subthemes were analysed and four themes emerged (Table 2). The comprehensive understanding of the memories of being injured and the trajectory of care is illustrated in a figure (Figure 1).

**Table 2:** Second structure analysis, with subthemes and themes

**Subthemes**	**Themes**
To be in a floating existence	Being connected to a surrealistic world
Changes of plans	
To be in an unreal world	
	
To be injured	Being connected to a injured body
To be enduring	
To be in No Mans' Land	
	
To receive attention	Being connected to care
To be taken care of	
To be cared for	
	
To be loved	Being connected to gratitude for life
To win life back	
To be in acceptance	

### Memories from the scene of the accident

#### To be in a floating existence

The event of the accident was something unexpected which caused panic and fear. When they were injured the informants had memories of their lives being rewound and at the same time that time was slowing down.

" I drove off the motorway and the car started skidding. A thousand thoughts came and went. I saw my whole life in that second, just as when you rewind a tape quickly." (15)

This timeless world was unpredictable and there was a lack of understanding of what was happening. When the body was disabled, they remembered that checking for paralysis and injuries was their first reaction when experiencing pain and discovering that legs and arms were in the wrong position.

" I understood it when my arms were hanging straight down that....and I could see that my hands were broken and that my arms were broken too." (8)

They also remembered a fear of dying and that their existence was in danger. The informants also experienced insecurity when thinking of their children's future, but they could not do anything; just trying to cope with it.

Those informants who had amnesia from the scene of the accident were told from family and friends what had happened. There were feelings of curiosity and sadness over not knowing but they chose to put it behind them.

"When my family and the work mates told me, I became curious and that surprised me. I also felt a little sad but what's happened has happened. I put it behind me. It's 'Water under the bridge'." (17)

#### To receive attention

The informants remembered that they received attention, help and kindness from other people who talked to and cared for them. When the paramedics arrived, there was a feeling of relief and trust even if they did not immediately understand why the paramedics had arrived.

"I know that this man told me to lay still. He'd already called the ambulance. I thought what for? It was just that it was bit hard to breathe and I thought that I'd better get to work." (1)

When realising that the paramedics' concern was aimed at them, the understanding that they had been in a serious accident became evident. The informants remembered that the paramedics warmed up their bodies, assumed responsibility and made them feel secure and safe.

"One minute after he'd called the emergency services, a nurse came. She told me to lie still and that she'd take care of everything. All I remember is her face above me. She had blonde, curly hair." (10)

### Memories from the scene of the emergency unit

#### Changes of plans

After the arrival at the emergency unit the informants remembered longing for their loved ones to come and pay attention to them due to their feeling of loneliness. They wanted to tell them about the changes of plans and trouble that they had brought about for others due to the injury.

"I was relieved when he finally came. I said: I'm sorry for ruining our holiday." (1)

The informants recalled that their family became worried when they received the message from the hospital about the accident. In those cases when the family or a particular person was not reachable, the informants felt insecure and lonely.

"I cried a lot in the emergency. It was the first Christmas that my children weren't at home. They were abroad with their dad and I couldn't get hold of them. My mother was away and my boyfriend, who I'd been with, had stayed at the party and didn't know what had happened. I felt pretty lonely. Yes, I did." (15)

#### To be taken care of

The memories from their arrival at the emergency unit were memories of security but also of anxiety and fear and they felt forlorn. However, the patients also talked about being taken care of by the emergency team. The informants remembered that many people were around them and the rapid pace; everyone doing what had to be done. Some staff members comforted them with talk about calling their loved ones and some panicked them with vague answers about their uncertain outcome. Patients remembered being stripped when their clothes were cut into pieces, something which resulted in a feeling of losing identity, shame and embarrassment.

" They opened the gates and there were a lot of people standing in there. That partly made it feel safe, but it was still scary. It became such a big deal. Then they moved me over to another stretcher with a lot of lamps on it. They cut my clothing into pieces and everybody did their thing, even though there were so many of them." (5)

### Memories from the scene of the Intensive Care Unit

#### To be injured

Patients had memories from the ICU stay of being stuck to a disabled and immobile body which was not functioning and also from not always being able to communicate verbally.

"The worst was being unable to speak. My family came to visit and I couldn't say anything. I spoke, but nothing came out. I wanted to talk about how things were at home." (3)

The first memories for participants with amnesia were fear when they woke up at the ICU and did not understand anything.

"I woke up at the ICU and realized that both my arms and my legs were a wreck, I didn't understand what had happed to me...I got afraid..." (7)

The patients remembered a lot of pain, a feeling of panic and thoughts about the risk of dying as they could not predict their condition, nor understand what had happened to them.

"I really never knew what the next day would be like and I got worse several times during my stay there. I thought I was going to die. " (8)

Those patients who had amnesia in the ICU narrated that it did not matter to them whether they recalled the ICU or not. Some even said that it was good not to remember the event of the injury and the ICU stay because they were afraid of the consequences this might have on their present life.

"When Mummy told me I thought that she was exaggerating but when the nurse told me I thought that Mummy probably hadn't really exaggerated. It was probably true all of it and as it was no pretty sight, it's better not to remember it." (18)

On the other hand, it was important to the patients to know if they had been on mechanical ventilation or not, and how injured they were, as this knowledge was essential to their perception of the severity of their injuries.

"What you don't remember in a way hasn't really happened. So it would have been good if the nurses had taken pictures of me so I could have seen how badly hurt I was – was it really that bad? Then you feel afterwards that you've got a second chance on life." (10)

The patients also recalled feeling vulnerable and exposed when unknown people knew more about them than they did themselves.

" I expect that I got the best care possible. Everyone told me their name but they were so many people and it felt unpleasant that everyone knew something about me but I had no idea who all these people were." (15)

#### To be in an unreal world

Patients also recalled experiences of being unable to reach other people, as if being in a bubble. They heard people calling their names to get their attention but were not able to communicate with them. This was connected to memories of crying and it made them feel as though they were outsiders.

"It felt as if I didn't see them around me. I heard them talking to me, but it didn't feel like I saw them; I just lay there crying." (16)

Those patients with delusional memories from the ICU had experienced, for example, hearing animal noises, being chased by insects and machines or being afraid of people that were sitting on the ceiling above their beds. For one patient the delusions was so real that she had named the persons that continuously occurred in the hallucination, there were one good pal and one bad and she felt protected by the good one.

"The hallucinations were unpleasant, but I'm still grateful to 'Michael' who was sitting above my bed. He was there for me, and helped me with those nasty people. He defended me when the others were sitting there throwing stuff at me. 'Daniel' threw pieces of paper at me. I was a bit angry with 'Michael' as well, though. Why didn't he tell them off more forcefully so that they would've gone a bit earlier? I have told everybody about this; they just laugh." (9)

Patients also experienced good dreams, for example with flowers and beautiful colures and of making a journey with the staff.

"I thought that it was the millennium, New Years Eve,...... I have been thinking a lot about this. I was flying in a helicopter and landed in my brother's garden. I heard voices very clearly. It was my mother, by brother, my sister-in-law and the nurses that I had met. It was all very weird – I thought that it was so kind of them to fix this millennium party just for me." (2)

There were also nightmares in which they for example were chased on their way home, or dreaming that death paid them a visit with fellow patients got killed. There were also memories of feeling fear and doubts and of being embarrassed about having been in this unreal world where they had no control. When telling their family and friends about these delusional memories, they experienced that they were not taken seriously. Remembering the time in the ICU with the delusional memories not knowing what was real, created uncertainty and shame.

"It's a bit disturbing. What is real and what isn't? Most of it I know isn't for real. There was no Michael up there in the ceiling. No, but I have really thought about that – how it all looked and where I lay and... I wonder what they thought about me. They must have thought that I was completely nuts...... I feel quite ashamed." (9)

#### To be cared for

Patients' memories of experiences in the ICU also consisted of a gratitude for life and how lucky they had been and that they had been saved. Friendly nurses were recalled who comforted them during sad moments and who understood their needs. There were also memories of caring activities that gave them physical comfort and a sense of well-being.

"I remember something I thought was so wonderful; the first time I could take a shower. Because I was in a lot of pain, they wheeled in a table to shower me. The nurses had rain gear on and I was lying there being showered. It was so nice. It was great to have this experience in the middle of all the misery." (2)

On the other hand, some patients also remembered that their self-esteem disappeared during the stay at the ICU; they felt vulnerable having to rely on others and not being able to take care of themselves.

"They had to change my diapers. It was unbelievably hard. The poor people working there... One's self-esteem disappeared." (8)

#### To be loved

In the interview text, there were also examples of good memories of visits from people close to them, for example the touch and the strength they received from a visiting relative. The patients still remembered feeling safe and connected to the world outside of the ICU when their loved ones visited them. They also felt loved and time passed more quickly during the visit.

"She was there every day and that felt good. It was nice to have someone, and Mom is always Mom. I felt quite small at times." (1)

On the other hand, patients also had memories of their own concerns for the family; not knowing if the family was capable to cope with all the trouble, worries and concern of what their injury had brought about. Thus, when family members arrived, the patients admitted to pulling themselves together, showing that they were able to cope and pose a better condition than they really were.

"Next time I woke up, my husband and my children were there at ICU so I pulled myself together for my children's sake so that they shouldn't see that I was that badly hurt." (5)

### Memories from the scene of the nursing ward

#### To be enduring

The patients stated that they remembered the care period following the ICU stay as difficult. There were also memories and thoughts of just how close to death they had really been. The injury had resulted in bodily discomfort and disabilities, which had raised an understanding of how injured they were. A few patients recalled some insulting situations and comments from the staff and physicians about their disabilities and said that they had felt abandonment when not receiving any alleviation of the pain. This resulted in strong feelings of worthlessness which made them unable to believe in their own capacity. The patients remembered that the conversation with the staff about their future situation came too early in their recovery, and that they wanted more empowerment.

"The information I was given in the beginning was about me probably not being able to speak or work. I was maybe going to be confined to a wheel chair. That affected me a lot...you have to look for the possibilities instead of the limitations... see the person who seeks care instead of the patient." (17)

#### To win life back

Patients described their memories of the transfer from the ICU to the ward. They received more privacy but less attention, realizing that it was up to them to get well. The memories from this period concerned exercise, putting up goals, feelings of insecurity and a struggle to get used to their injured bodies.

"They told me to retrain, because I could never return to my old job. I then decided that I would be able to walk by Easter and work by Midsummer. In the beginning it was only sporadic training because they wanted to see how much I could take. Then there was training both mornings and afternoons, so I cut out the therapy to go training instead." (3)

The patients struggled to win their lives back, and return to a life that felt meaningful. In some cases they did not want to remember the accident; it had to be left behind. For those with several weeks of amnesia, memories from visits of friends and family at the ward and special events were remembered. To be in a fellowship with other patients became important. They talked and went through the injury again and again with other patients and their friends, remembering having a need to do so.

"You talk with all your friends who visit and this way you go through the accident again and again. It's a bit like therapy when you get to talk about it. I was lucky because I shared a room with a girl at my age. We talked a lot, laughed and were being silly. Sometimes they (the staff) came in asking what we were doing." (1)

Some patients remembered feelings of embarrassment when they had been talking nonsense and when memory lapses occurred in the conversation. The good memories of this period of care involved their striving to reach the goal to go home and get well, encouraged by their relatives.

"I wanted so badly to get well for his sake and for my son's sake. They (the husband and the son) said that I would get well." (2)

### Memories from the scene of coming home

#### To be in No Man's Land

The informants recalled arriving home to a reality were they did not fit in, resembled being on their own in No Man's Land, and they experienced being dependent and a burden to others. Signs of encouragement from others were interpreted as signs of regret and of life being over.

"When I came home from the hospital in my wheelchair it was warm so I sat outside. Flowers arrived and stuff and I thought, 'Is it somebody's funeral?' That was hard." (12)

The informants realised that they had to create their own existence, realizing that their bodies were injured and that life was forever changed. Further, some also remembered that the community health care service did not meet their needs and did not sufficiently assist them. For example, a man returning home in a wheelchair tried to convince the community health care service that it was possible to have a ramp for the wheelchair out into the garden. They did not believe him until he showed them that it was possible for him to get through the door with the wheelchair into the garden.

"I didn't get the disability-aids I needed and when you got out of the hospital it was like you were regarded as a 'closed case' that you no longer needed any help." (3)

A woman had cats at home and when she arrived home she could not walk or bend her back so it was impossible for her to feed the cats. The community service did not assist her to find solutions. Hence, these informants felt that they were of no interest, which increased their feeling of being a burden to their families. They felt that the caregivers had abandoned them.

"All the time while I was so ill I constantly worried about whether my husband would have the strength to cope with me. If I didn't get well, I simply couldn't let him carry the entire burden." (2)

At the time of the interview, the informants expressed experienced limitations both socially and professionally after returning home. The informants felt inactive and forgotten; not able to socialise with friends on the same terms as before. Even if struggling hard, some experienced failure as they did not recover and their functional activities were not improved. The expectations of not being able to fully function in the way they wanted any more, made them feel worthless and of no use which lead to feelings of wanting to give up, just exist and drift.

"I cannot do anything. I used to have a boat but I can forget that now. I can't jump off boats and things like that that, nor can I walk in the woods. Now I only drive around in the car, doing nothing. Yes, I do absolutely nothing. I have put on 15 kg since the accident happened. Everything is so different, I cannot play any sports or play football......it is all crap." (4)

After the arrival home, the informants also suffered extreme tiredness which caused the previous nightmares and bad memories from the injury to return.

"One afternoon a couple of days after I come home from the hospital, those dreams came back again. It felt just like in the ambulance. When I fell asleep, they were there. It happened last week again... I can lie down several times a day....my entire body feels tired." (5)

Some informants lost their sense of smell and taste. They sometimes had sensations of disappearing from reality which were frightening, and also not recognising themselves. When the recovery took time, they almost did not have the strength to go on and fight any more. When the expectation to recover failed, the wish to give up grew stronger.

"I tried to struggle on as long as I could. Then I realised I wouldn't get any better. Since then I've gotten worse. I thought I would get well. The doctor said, 'See it as premature ageing." (13)

This waiting for recovery changed them and made them feel strange. They did and said things not really meant and were suffering from the fact not being able to fully recover.

#### To be in acceptance

On the other hand, there were also other recollected experiences. Many of the informants' memories of arriving home and the following period were focussed on the future and on putting the trauma behind them. The delusional memories did not bother most of them any more. Work, if only for a few hours a day, and the will to recover became extremely important for all.

"We'll see if I can manage to work, I plan to go in a couple of hours a day at first, and then work part time and so on... If I manage that I can call my self healthy." (11)

Life had become different and they realised the meaning of being given a second chance, hence showing a humble spirit, not taking life for granted. They felt grateful for having survived and realised that things could have been worse.

"I enjoy life more than before and I'm grateful to be alive." (16)

After arriving home, their family, friends and superiors at work gave them support and they felt accepted and included. Feeling a will to manage and survive and in some ways accept the situation became stronger even if their new lives consisted of limitations. They knew that neither their lives nor themselves would ever be the same as before the accident.

"I'm so used to being the way I am now. I'm healthy in my own way now, but I'll never be as before. I will never experience that time again. I'm a new man now. I will have to live with that." (18)

### Themes of memories

Four themes emerged when analysing the twelve subthemes; a surrealistic world, an injured body, care and gratitude for life (Table 2). When the injury occurred the "surrealistic world" appeared with memories of being in a floating existence, changes of plans and being in an unreal world. This world was unfamiliar and it was experienced as uncontrollable.

To be "connected to an injured body" with memories of being injured, enduring, and being in No Man's Land limited their lives. To exist in this world of trying conditions implied that life would forever be different and the future more uncertain.

At the same time they were "connected to care" with memories of receiving attention from others, being taken care of and being cared for. This care made them realise that people are responsible for each other, and the informants felt comforted but also vulnerable.

When the informants felt "connected to gratitude for life", they had memories of being loved and had the strength to struggle to win life back, and to accept that their lives had become different after the injury. This helped them and gave them courage to live and move on even if the future in many ways was felt to be uncertain.

### Comprehensive understanding

The different memories were balancing each other during the trajectory of care before, during and after the ICU stay. Bad memories of a surrealistic world and of being injured seemed to connect the individual to feelings of hopelessness and worthlessness. This could be counteracted by good memories of care and of a gratitude for life.

Some informants moved on, experiencing a gratitude for life despite their limiting injured body and an uncertain future, while others had difficulties to move on because they felt connected to an injured body with limitations and consequently their future seemed more uncertain (Figure 1).

## Discussion

The purpose of this study was to acquire a deeper understanding of patients' memories of being injured and the trajectory of care before, during and after the ICU stay. The findings show that the injury is not a single event for the patient; it is the start of several events within a trajectory of care and a change of life for the survivor.

When the informants were looking back describing their experiences, they were living with both good and bad memories from the past and with a future that was felt to be uncertain. The trajectory of care drifted between these good and bad memories. The surrealistic world with bad memories was frightening, but there were also pleasing delusional memories such as travelling with the staff who where not causing fear. These good-nature delusions may have given some protection from the outside world when the injured person was unable to deal with all the terrible facts. These peculiar experiences can hold a positive potential [17]. There were two informants with smaller injuries who told us that the nightmares from the ICU returned when they suffered from stress or got tired at home. It is possible that the two patients may have symptoms of post traumatic stress disorders (PTSD). In a study by Jones et al [18] delusional memories were a precipitant for PTSD. In contrast to this, in our study the delusional memories from the ICU did not seem to bother most informants after returning home. However informants with delusional memories still remembered them clearly, which is in accordance with findings from a recent study [19].

The factual, emotional and delusional memories of the accident and care were at the time of the interview 2–3 years after the injury still important for the informants, but they also struggle with many other problems. There were problems with returning back to work, with disability not being able to do things in the same way as before and there were still memories of the accident with fear evoking thoughts about their own mortality.

Other bad memories were the injured body with its limitations which made them feel insecure and frightened. To be injured is a threat to one's existence. How human beings respond to threats is described by Morse in five different steps [11] in the extended theory "Responding to threats to integrity of self". The first step is vigilance, followed by enduring to survive, then enduring to live, suffering and finally, learning to live with the altered self. In the present study, we found that all these steps [11] were identified in the trajectory of care. The vigilance and enduring to survive were shown at the scene of the accident, in the emergency unit, and at the ICU when the informants related their memories from these places where their lives had been in most danger. During the stay in the nursing ward and when the patients realised how injured they were, the focus was on trying to bear it and learning to take it [11], in other words enduring to live. The suffering and learning to live with the altered self started when they had returned home. The trauma and the recovery from it has also been described as a journey with an event, fallout, and moving-on [3]. In our study we recognised the three phases of the journey to recovery.

However, we also found that the trajectory of care held different possibilities of and preconditions for care which could produce good memories, something which seemed to be of importance for the injured persons' ability to move on and feel well despite their injured bodies and limitations. This gave us the opportunity to reconsider the care activities within their trajectory.

At the scene of the accident, and in the emergency unit, many patients remembered friendly and caring actions with comforting talk, something which also has been studied earlier [20]. Our study also shows the importance of putting great effort into locating the relatives as quickly as possible. Uncaring activities in the emergency unit, such as when their clothes were cut into pieces and when they received information about an uncertain outcome, made the individuals feel ashamed and insecure. These actions are in most situations necessary, but they can be problematic when causing a loss of identity and anxiety in a person who already is in a vulnerable situation. The importance of caring encounters and meeting the patients' psychological needs in the emergency care has been described by Wiman & Wikblad [21].

In the ICU, care actions such as being considerate and wanting to understand the patients' unreal world with its delusional memories, fear and problems of not knowing what is real need to be improved. The patients' need of true presence from the nurses to feel safe and understood, has also been articulated earlier [22-24]. Another care activity which emerged in our study was the facilitation of communication between patients and family during visits in the ICU. The patients often had communication problems due to mechanical ventilation and sedative medication [25]. The family and loved ones are the ICU patients' lifeline to reality and the nurse should draw the family's attention to this [26].

In the nursing ward, caring actions consisted of facilitating a fellowship with other patients with trauma, and of empowering the individuals to move on. The change in the level of care from the ICU to the ward was obvious with both negative and positive memories as a consequence. The transfer from the ICU can be problematic [27,28] and in our study the patients realised that they had to find their "new lives" by exercising and getting used to their new body.

After returning home, it was found that informants felt abandoned by the health care system because nobody seemed to be responsible for their rehabilitation and care. The informants and their family seemed to need more support. Jones et al [29] have described different caring programmes, such as rehabilitation programmes after the ICU care. We suggest that the injured person should receive guidance for a long time, even years, after the trauma, this has also been articulated earlier [30]. Having a supporting family and friends was the most important part of being connected to gratitude for life and to move on, focussing on the future. This study supports Maddox et al [8] findings of the relatives' importance to the patients. Those informants who were not able to move on seemed to be without caring families, and their injured bodies seemed to bear an injured spirit, thus becoming an injured person. When bad memories are balanced by good ones, there are more possibilities to move on towards an uncertain future after the injury and the ICU care.

We considered it important to focus on memories from different scenes from the trajectory of care. Therefore it was of interest to gain knowledge about the patients' memories from events of this trajectory, because such knowledge could lead to a development of caring actions that patients will remember, and remember favourably. In this study, one important finding was the informants' memories of knowing that other people, such as care providers, family, and "the man on the street" took care of them when they needed it, giving them confidence in knowing that "no man is an island". Being in need of help can be seen as an ethical demand [31]. This demand was here implicit and brought about by the informants putting their lives in the hands of the medical staff, trusting the notion that each human being is considered to be responsible for the "other". This is based on an assumption of a spontaneous manifestation of life involving confidence, trust and love. These traits are per se ethical and characterised by an idea of reciprocity between human beings. Human life implies that trust between individuals is elementary. To show trust means to expose yourself. Should a person remain unaffected by this exposure, the trust has been abused. If the staff appeared unaffected the informants felt offended or abandoned by the staff. Such activities could be regarded as uncaring.

Good caring actions as well as family support resulted in that patients felt cared for and safe, which made them realise that they fitted in just as they were. It seemed to be important for the informants in this study to have good memories of care and of human love to counterbalance those memories of the surrealistic world and of living with an injured body.

In the present study only three of 18 patients were suffering from total amnesia for several weeks. Amnesia can be rather common for patients with trauma [32] but in our study there were also patients without mechanical ventilation. This might be the reason for the better recollection and that the delusional memories were still very clear for all. For those patients in this study who had amnesia it was a relief not to remember the accident. However it was important that family members and nurses told them what had happened when they were in care. These stories from others enabled them to create their own history around the trauma and the care, as well as letting them know that they were loved. This became these individuals' being in the world [14].

### Methodological considerations

We had to consider some methodological issues. Our aim was not to seek the ultimate interpretation, but to seek one out of many possibilities related to what is said in the text. We also sought the most useful understanding in relation to our own pre-understanding. To broaden our pre-understanding, and hence the possibility to interpret the text in a relevant manner and reach new understanding, we used previous theoretical concepts and models [3,10,11,31]. When we interpreted the text, we also looked for sub-themes that were contrary to our main interpretation. Finally, it is only when the reader of this research article can make sense of the findings and integrate them in his or her own world that they can be used for improving care [13].

## Conclusion

This study highlights memories following a trauma; how it was to be injured and cared for and then returning back home. There are both good and bad memories during the trajectory of care. These memories may balance each other. This balance of memories allows the person to move on, not being captured in a surrealistic world with an injured body. Finally, the most important thing during the trajectory of care is to support the presence of caring relatives and caring nurses who help the patient concentrate on acceptance and on feeling of gratitude for life. When bad memories are balanced by good ones, there are more possibilities to move on from the injury and the ICU care, despite an uncertain future.

## Abbreviations

ICU: Intensive care unit; ISS: Injury severity score.

## Competing interests

The authors declare that they have no competing interests.

## Authors' contributions

MR contributed in all stages of the research: design of the study, conducted the interviews, analysing and in writing the bulk of the paper. IB contributed in the design of the study, analysis and writing. KP contributed to the writing. All authors have read and approved the final manuscript.

## Pre-publication history

The pre-publication history for this paper can be accessed here:


